# Are Reductions in Population Sodium Intake Achievable?

**DOI:** 10.3390/nu6104354

**Published:** 2014-10-16

**Authors:** Jessica L. Levings, Mary E. Cogswell, Janelle Peralez Gunn

**Affiliations:** Division for Heart Disease and Stroke Prevention, National Center for Chronic Disease Prevention and Health Promotion, Centers for Disease Control and Prevention, 4770 Buford Hwy NE, Mailstop F72, Atlanta, GA 30341, USA; E-Mails: mcogswell@cdc.gov (M.E.C.); jperalez@cdc.gov (J.P.G.)

**Keywords:** sodium, salt, blood pressure, population salt reduction, sodium reduction in the food supply, salt intake and health, salt taste preference

## Abstract

The vast majority of Americans consume too much sodium, primarily from packaged and restaurant foods. The evidence linking sodium intake with direct health outcomes indicates a positive relationship between higher levels of sodium intake and cardiovascular disease risk, consistent with the relationship between sodium intake and blood pressure. Despite communication and educational efforts focused on lowering sodium intake over the last three decades data suggest average US sodium intake has remained remarkably elevated, leading some to argue that current sodium guidelines are unattainable. The IOM in 2010 recommended gradual reductions in the sodium content of packaged and restaurant foods as a primary strategy to reduce US sodium intake, and research since that time suggests gradual, downward shifts in mean population sodium intake are achievable and can move the population toward current sodium intake guidelines. The current paper reviews recent evidence indicating: (1) significant reductions in mean population sodium intake can be achieved with gradual sodium reduction in the food supply, (2) gradual sodium reduction in certain cases can be achieved without a noticeable change in taste or consumption of specific products, and (3) lowering mean population sodium intake can move us toward meeting the current individual guidelines for sodium intake.

## 1. Introduction

The vast majority of Americans consume too much sodium, primarily from packaged and restaurant foods [1,2]. The evidence between sodium intake and direct health outcomes indicates a positive relationship between higher levels of sodium intake and increased cardiovascular disease risk, consistent with the relationship between sodium intake and blood pressure [3]. In the United States, professional health organizations, including the American Heart Association, the American Medical Association, and the American Public Health Association, as well as recommendations from the 2010 *Dietary Guidelines for Americans* and Institute of Medicine (IOM) support dietary sodium reduction to prevent and control high blood pressure. Further, *Healthy People 2020* aims to “Reduce consumption of sodium in the population aged 2 years and older” from a mean of ~3600 mg per day from all sources to 2300 mg per day; about a 40% reduction over 10 years [4,5].

Despite communication and educational efforts focused on lowering sodium intake over the last three decades, data suggest average US sodium intake is remarkably elevated and consistent, leading some to argue that current sodium guidelines are unattainable [6]. The IOM in 2010 recommended gradual reductions in the sodium content of packaged and restaurant foods as a primary strategy to reduce US sodium intake, and evidence since that time suggests gradual, downward shifts in mean population sodium intake are achievable and can move the population toward current sodium intake guidelines. In addition, new modeling estimates indicate even moderate reductions in average population sodium intake can lead to reduced hypertension and prevent deaths due to heart disease and stroke [7].

## 2. Can Reducing Sodium in Packaged and Restaurant Foods Reduce Population Sodium Intake?

According to the IOM and 2010 *Dietary Guidelines for Americans*, a reduction in the sodium content of foods in the marketplace is necessary to allow consumers to reduce sodium intake to less than 2300 mg or 1500 mg per day. Further, the 2010 *Dietary Guidelines for Americans* specifically state that gradual sodium reduction is a long-term goal, both to allow the food industry time to formulate new products and reformulate existing products and to give consumers time to shift their preferences toward lower sodium foods.

Given the current food environment, it is difficult for even motivated individuals to meet sodium intake recommendations [8]. Importantly, most Americans have exceeded sodium intake recommendations before adding salt at the table [9,10,11]. Sodium reduction can be difficult for the individual consumer considering it’s not only foods high in sodium that are main contributors to sodium intake, but also foods that are frequently consumed and have moderate amounts of sodium, such as breads and poultry [12]. Different brands of the same foods often vary widely in sodium content [13], and research has found that sodium levels differ for the same brand of specific foods sold in different countries [14,15] meaning in many cases manufacturers can produce foods with less sodium.

New evidence indicates: (1) with strategies to gradually reduce sodium in the food supply before the food reaches the consumer, significant reductions in mean population sodium intake can be achieved, (2) sodium content of many foods can be reduced gradually without consumers noticing the change in taste or compensating by changing consumption patterns, and (3) shifting mean population sodium intake can move us toward meeting the current individual guidelines for sodium intake.

## 3. Significant Reductions in Mean Population Sodium Intake Can Be Achieved with Gradual Reduction of Sodium in the Food Supply

Efforts in other countries demonstrate the effectiveness of population interventions aimed at reducing sodium in packaged foods on reducing population sodium intake. Webster and colleagues [16] recently reviewed salt reduction initiatives around the world and found that certain countries, including Finland and the United Kingdom (UK) experienced a reduction in population sodium intake resulting from working with the food industry to reduce the sodium content of packaged foods.

In Finland, the North Karelia Project began in 1972 as a national pilot and demonstration program for cardiovascular disease (CVD) prevention. At this time, salt intake was approximately 12 g/day (4800 mg/day sodium) [1]. The program aimed to reduce CVD risk factors in the population including smoking, elevated cholesterol, and elevated blood pressure. To assist in lowering blood pressure, salt reduction was one component of the program. Resulting from the multi-faceted program, sodium intake declined to 3900 mg/day for men and 2700 mg/day for women, and a 60% drop in coronary heart disease and stroke mortality occurred between 1978 and 2002 in Finland [1]. The salt reduction segment of the program focused on the following strategies: (a) clear population sodium reduction targets set by the Finnish National Nutrition Council, (b) regular monitoring of population salt intake, (c) mass media campaigns and education of healthcare personnel, (d) extensive involvement from stakeholders and the community, (e) voluntary collaboration by the food industry to reduce salt, and (f) mandatory warning labels for foods high in salt, leading to significant reductions in the average salt content of many food categories, including breads [17,18].

Similar to Finland, a main tenet of the UK sodium reduction initiative launched in 2003 also included targets for sodium reduction in the food supply and working with the food industry to gradually achieve those targets. Between 2000/2001 and 2011 population salt intake declined by 15% from 9.5 g to 8.1 g per day, which along with other lifestyle changes, produced parallel declines in mortality from stroke and ischemic heart disease [19]. However, it is important to note that baseline sodium intake in both Finland and the UK was significantly higher than in the US, which may facilitate earlier results in population reduction. Additionally, an analysis of the salt content of 18 different bread products (a primary source of sodium in the UK) sold in UK supermarkets in 2001, 2006, and 2011 found that mg sodium per 100 grams declined by 17% from 496, to 456, to 412 [20], respectively. In addition, the Irish Food Standards Agency set and achieved the following sodium reduction targets: 10% reduction in breads, 15% reduction in sauces, and 10% reduction in soups [21].

While no regulatory and limited voluntary efforts have been undertaken at the national level, to date, in the US to reduce sodium in the food supply, the Food and Drug Administration (FDA) and U.S. Department of Agriculture (USDA) solicited comments in 2011 “to obtain comments, data and evidence relevant to the dietary intake of sodium as well as current and emerging approaches designed to promote sodium reduction.” Also in 2011, FDA, USDA, and the Centers for Disease Control and Prevention (CDC) hosted a Public Meeting entitled, “Approaches to Reducing Sodium Consumption” to provide an opportunity for in person comments and discussion. Voluntary efforts to reduce the sodium content of packaged and restaurant food in the US are also underway. The New York City Department of Health and Mental Hygiene’s National Salt Reduction Initiative (NSRI), a partnership of more than 90 state and local health authorities and national health organizations, developed voluntary salt reduction targets for 62 packaged food and 25 restaurant food categories in 2012 and 2014. As of April 2014, 27 companies were committed to the initiative and some popular products already meet the targets indicating they are achievable. Other large retailers and restaurants are also vowing to reduce or continue reducing sodium. Walmart, one of the country’s largest food retailers, publicly committed to a 25% reduction in sodium by 2017 in many grain, meat, dairy, sauce and condiments, snack, and prepared food products. Walmart estimates that US adults would consume approximately 47 million fewer pounds of sodium each year if the company’s reformulations were adopted across the entire grocery industry. Darden, the world’s largest full service restaurant company, pledged a 10% reduction in sodium across its portfolio by 2016 and 20% by 2021.

## 4. Sodium in Certain Foods Can be Gradually Reduced without Affecting Taste or Changing Consumer Consumption Patterns

The top food categories contributing to sodium intake in the US are breads, cold cuts and cured meats, pizza, poultry, soups, sandwiches, cheeses, pasta mixed dishes, and meat mixed dishes [13]. Research suggests consumers do not notice sodium reductions of up to 20%, depending on the food product [1]. While an overall 20% reduction of sodium in foods could have a large impact on intake, it is recognized that the amount of reduction may need to vary by food category to avoid changes in consumer consumption patterns. In addition, salt taste preference may differ when foods are consumed as a component of another food, e.g., bread or cold-cuts as part of a sandwich, when products are more or less complex, and when there is strong brand loyalty and recognition.

In the US, bread, although moderate in sodium, is the top food source of sodium intake due to how much is consumed. Globally, studies suggest sodium reductions in foods moderate in sodium, like bread, and those considered to be salty, like cheese, can be achieved without a noticeable difference in taste. In regards to flavor and consumer acceptance, studies suggest significant reductions in the sodium content of various cheeses is possible without detection by consumers or trained tasters. Results of two recent US studies indicate sodium reductions of 8% in cottage cheese and 60% in cheddar cheese did not affect consumer liking or acceptance compared to higher sodium samples [22,23].

For dairy products such as low moisture mozzarella cheese, the use of a sodium chloride/potassium chloride mix had similar effects on the texture profile and microstructure, indicating the salt in this type of cheese also could be partially replaced by potassium chloride [24]. However, anecdotal evidence indicates that replacement of salt with potassium chloride in foods in general may not be ideal for specific brands of foods with a loyal consumer following since consumers would likely notice the change in taste. For breads, salt reductions of up to 30% were not detected in French breads by a panel of expert testers [25]. For brown bread, salt reductions of up to 67% did not impact consumption among consumers, indicating taste was still acceptable [26]. Consumers of white bread in which 30% of the sodium was replaced by potassium salts reported acceptability scores similar to the control bread [27]. Thus, a significant reduction in sodium, even when coupled with increases in potassium, could be achieved in different types of breads.

In addition, evidence also suggests that people will add back less salt with the salt-shaker after sodium has been reduced in packaged food [28,29]. One study found that on average, participants added back less than 20% of the sodium removed from the food when allowed unlimited use of the salt-shaker [29]. While gradual implementation has been suggested as a means to achieve sodium reductions in the food supply [1], few studies have tested gradual sodium reduction over a significant period of time to assess the feasibility as a long-term strategy. Further research will aid in the understanding of the impact of gradual reductions in sodium on consumer liking and acceptance over time.

## 5. Shifting Mean Population Sodium Intake Can Move US toward Meeting the Current Individual Guidelines for Sodium Intake

Data from INTERSALT indicate mean population sodium intakes have ranged from less than 200 mg/day in the Yanomamo Indians of Brazil to more than 10,000 mg/day in Northern Japan [30]. Usual long-term sodium intake also varies substantially among individuals with evidence suggesting at least some individuals have usual intakes <1500 mg/day and some >6000 mg/day. Importantly, a small downward shift, as little as 400 mg in average population sodium intake, could result in a large reduction in the proportion of individuals consuming excess sodium.

**Figure 1:**
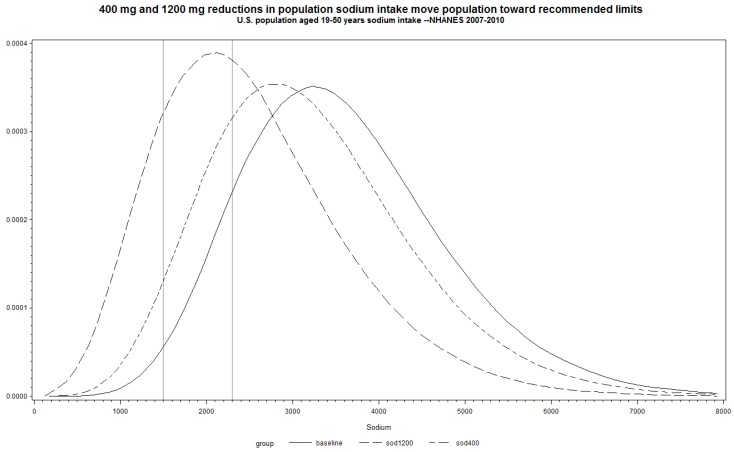
400 mg and 1200 mg reductions in population sodium intake move population toward recommended limits.

As an example, we estimated average usual population sodium intake using two days of 24-h dietary recall data from the National Health and Nutrition Examination Survey, 2007–2010 adjusting for within person variation in measurement error [31]. Reducing average intake among US adults age 19–50 years by 400 mg would shift the proportion of individuals consuming >2300 mg and >1500 mg from 89% to 79%, and 99% to 96%, respectively. If average sodium intake was reduced by 1200 mg, an even greater reduction in the percentage of the population consuming >2300 and ≥1500 mg would occur, decreasing to 54% and 84%, respectively (Figure 1). Thus, reductions in sodium intake, even as little as 400 mg, are related to large and meaningful changes in the proportions of individuals with usual intake above certain thresholds.

## 6. Conclusion

It is estimated over 75% of sodium intake comes from packaged and restaurant foods, not from salt added by the consumer, making it difficult for even motivated consumers to control their sodium intake. Although the 2010 IOM report on *Strategies to Reduce* *Sodium Intake in the United States* concluded that previous individual consumer-based efforts have been ineffective, the report identified the current food environment as the main barrier to these efforts. In addition, previous US efforts did not focus specifically on gradually reducing sodium in packaged and restaurant foods with attention to salt taste preference. Although the effect of gradually reducing sodium in packaged and restaurant foods on population sodium intake has yet to be determined in the US, both previous and newly emerging research indicate this approach is feasible. Gradual reductions in sodium across the food supply will help to shift population intake to a lower level, giving consumers more control over their personal sodium intake without compromising taste preferences.
